# Supplementary figures and images for: Comparative analysis of transcriptomic profiles among ascidians, zebrafish, and mice: Insights from tissue-specific gene expression
Source: PLoS One. 2021 Sep 24;16(9):e0254308. doi: 10.1371/journal.pone.0254308 (PMC8462739; doi:10.1371/journal.pone.0254308)

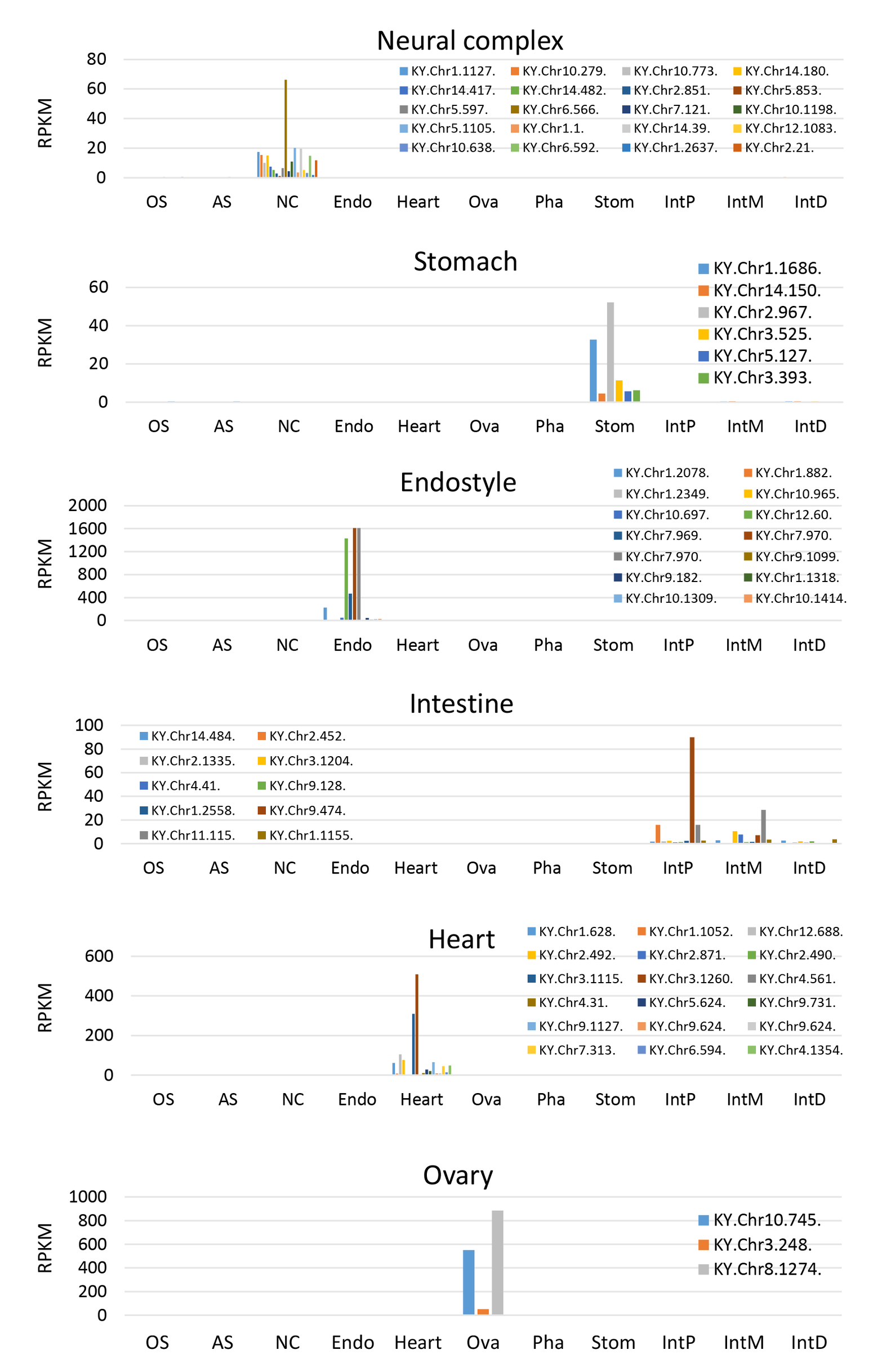

Supplement: S1 Fig — The tissue specificity of the TSGs previously reported by Shoguchi et al., 2011 [27] was confirmed in the current RNA-seq data. AS, atrial siphon; Endo, endostyle; IntD, distal intestine; IntP, proximal intestine; IntM, middle intestine; NC, neural complex; OS, oral siphon; Ova, ovary; Pha, pharynx; Stom, stomach. (TIF) [file pone.0254308.s001.tif]

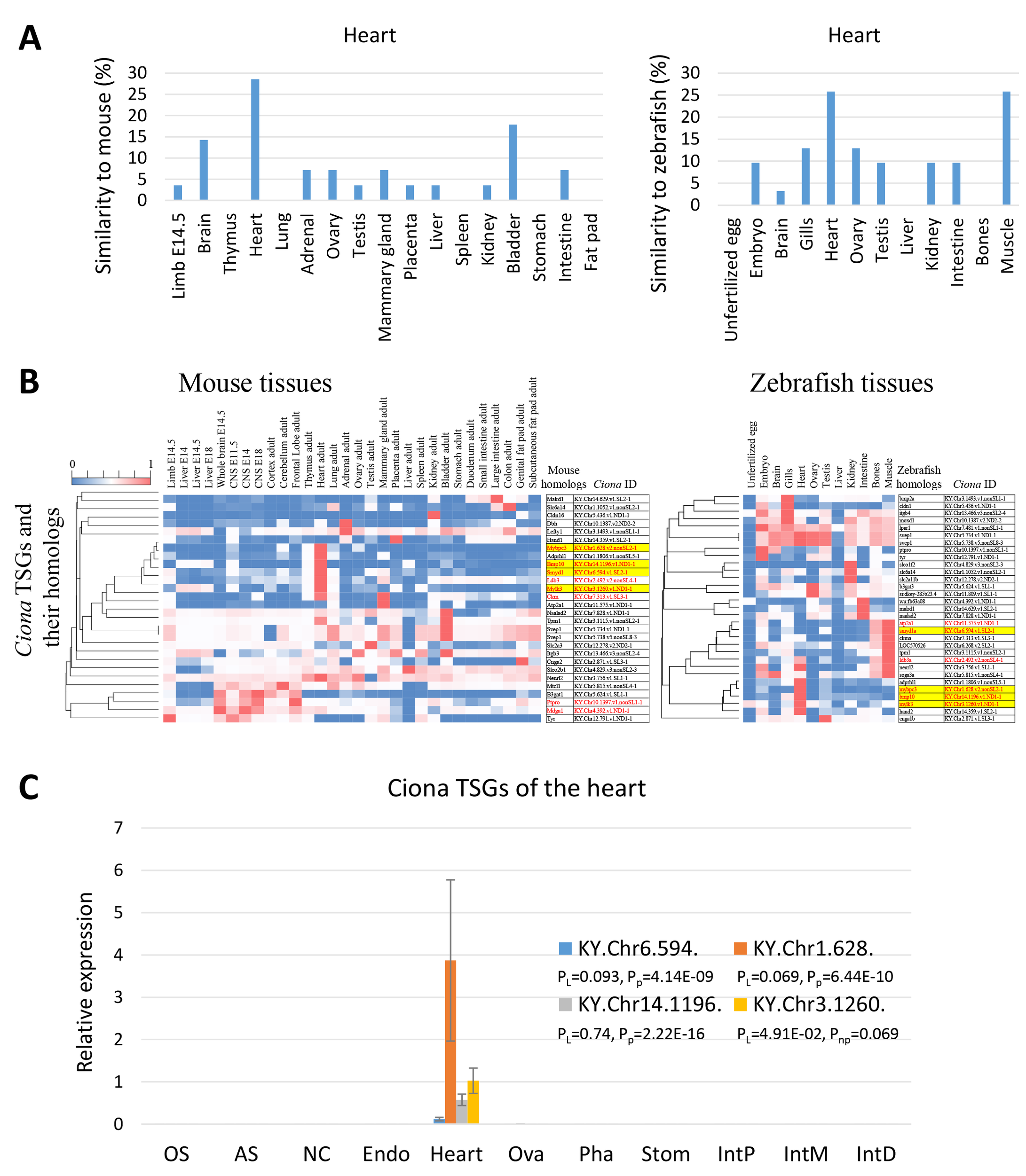

Supplement: S2 Fig — (A) Similarities between Ciona heart and mouse (left) or zebrafish (right) tissues were calculated as in Fig 3A. Approximately 30% and 25% of the homologous genes were highly expressed in the mouse heart and zebrafish heart and muscle, respectively. (B) Clustering by tissue distribution of the homologous genes in mice and zebrafish. The heat maps are shown as in Fig 3B. (C) The heart-specific expression of Ciona TSGs was confirmed by qRT-PCR (n = 3–4). Data are presented as in Fig 3C. (TIF) [file pone.0254308.s002.tif]

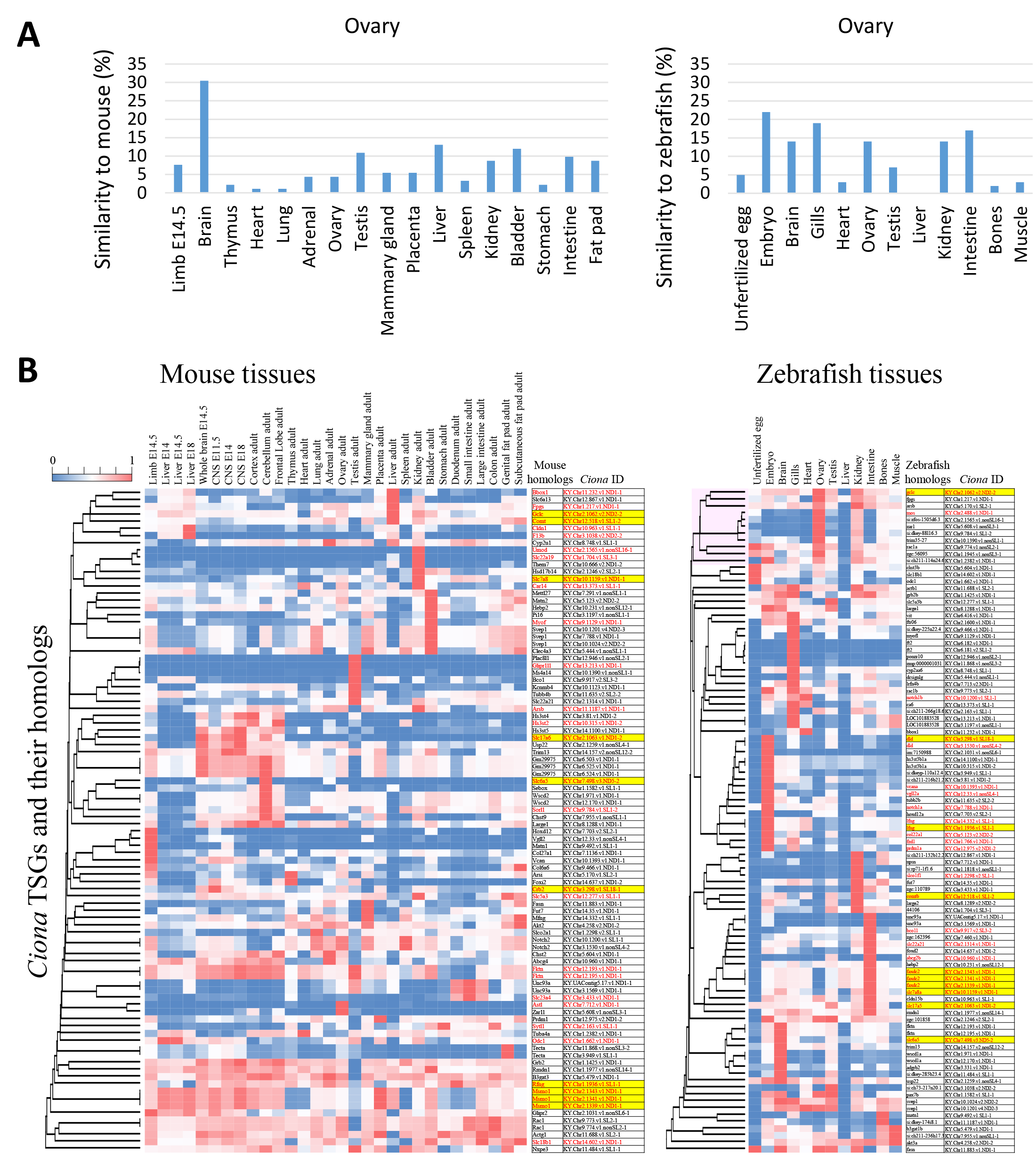

Supplement: S3 Fig — (A) Similarities between Ciona ovary and mouse (left) or zebrafish (right) tissues were calculated as in Fig 3A. (B) Clustering by tissue distribution of the homologous genes in mice and zebrafish. The heat maps are shown as in Fig 3B. The zebrafish-ovary cluster is shown in pink. (TIF) [file pone.0254308.s003.tif]

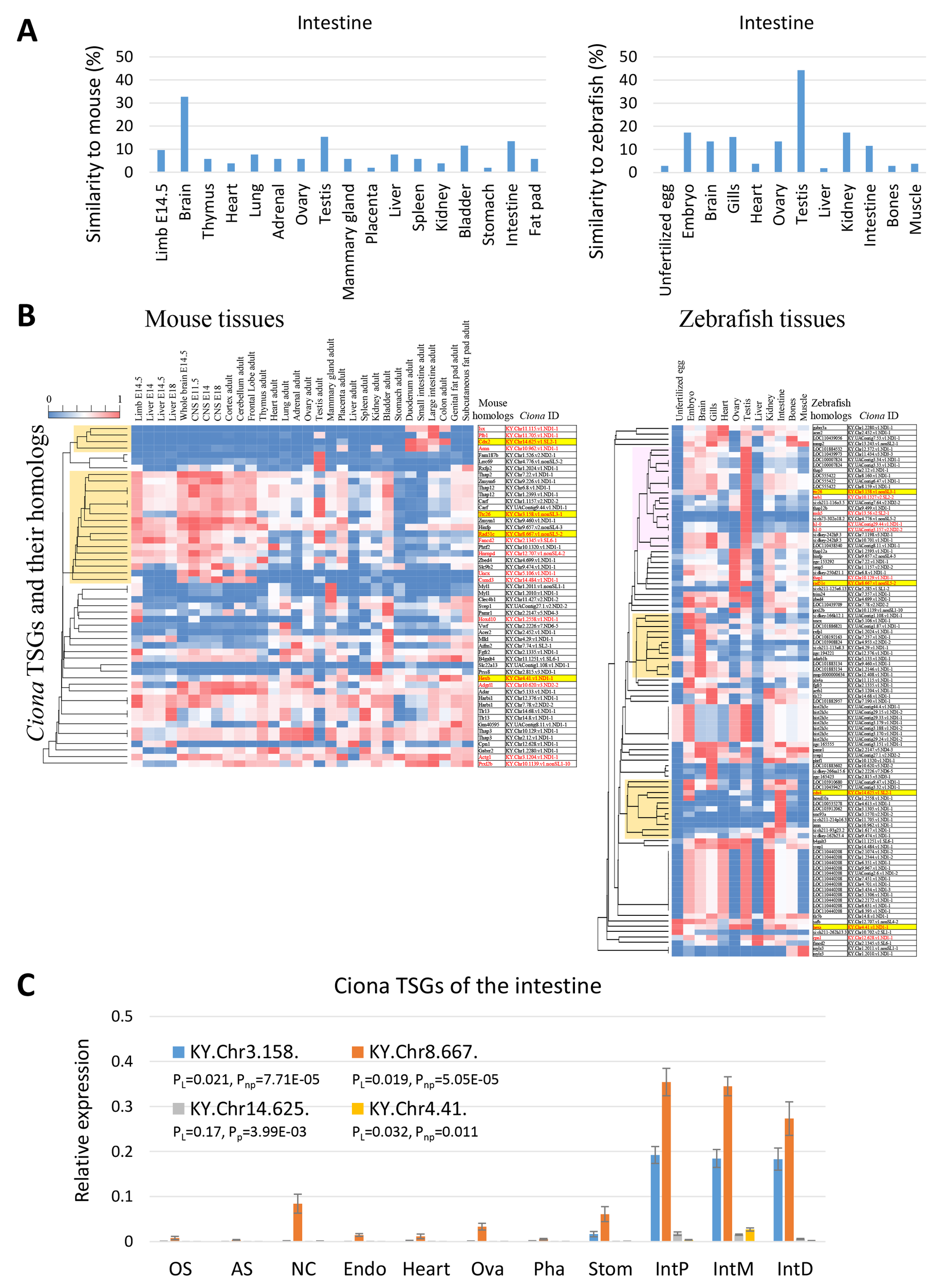

Supplement: S4 Fig — (A) Similarities between Ciona intestine and mouse (left) or zebrafish (right) tissues were calculated as in Fig 3A. (B) Clustering by tissue distribution of the homologous genes in mice and zebrafish. The heat maps are shown as in Fig 3B. The clusters of highly expressed genes in the mouse brain and intestine are shown in orange, and that of the zebrafish testis is shown in pink. (C) The intestine-specific expression of Ciona TSGs in the heart were confirmed by qRT-PCR (n = 3–4). Data are presented as in Fig 3C. (TIF) [file pone.0254308.s004.tif]

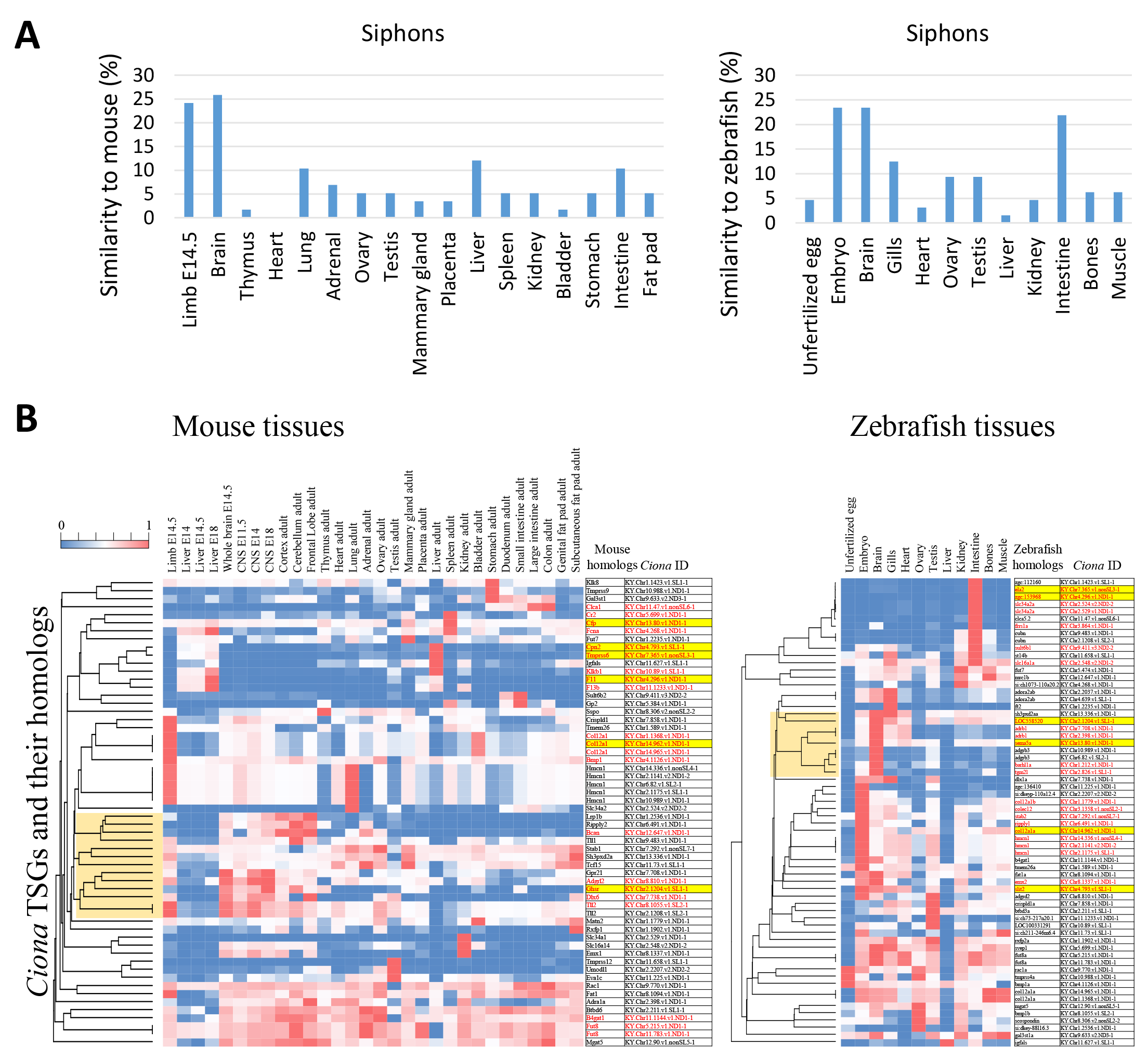

Supplement: S5 Fig — (A) Similarities between Ciona siphons and mouse (left) or zebrafish (right) tissues were calculated as is Fig 3A. (B) Clustering by tissue distribution of the homologous genes in mice and zebrafish. The heat maps are shown as in Fig 3B. The clusters of highly expressed genes in the vertebrate brain are shown in orange. (TIF) [file pone.0254308.s005.tif]

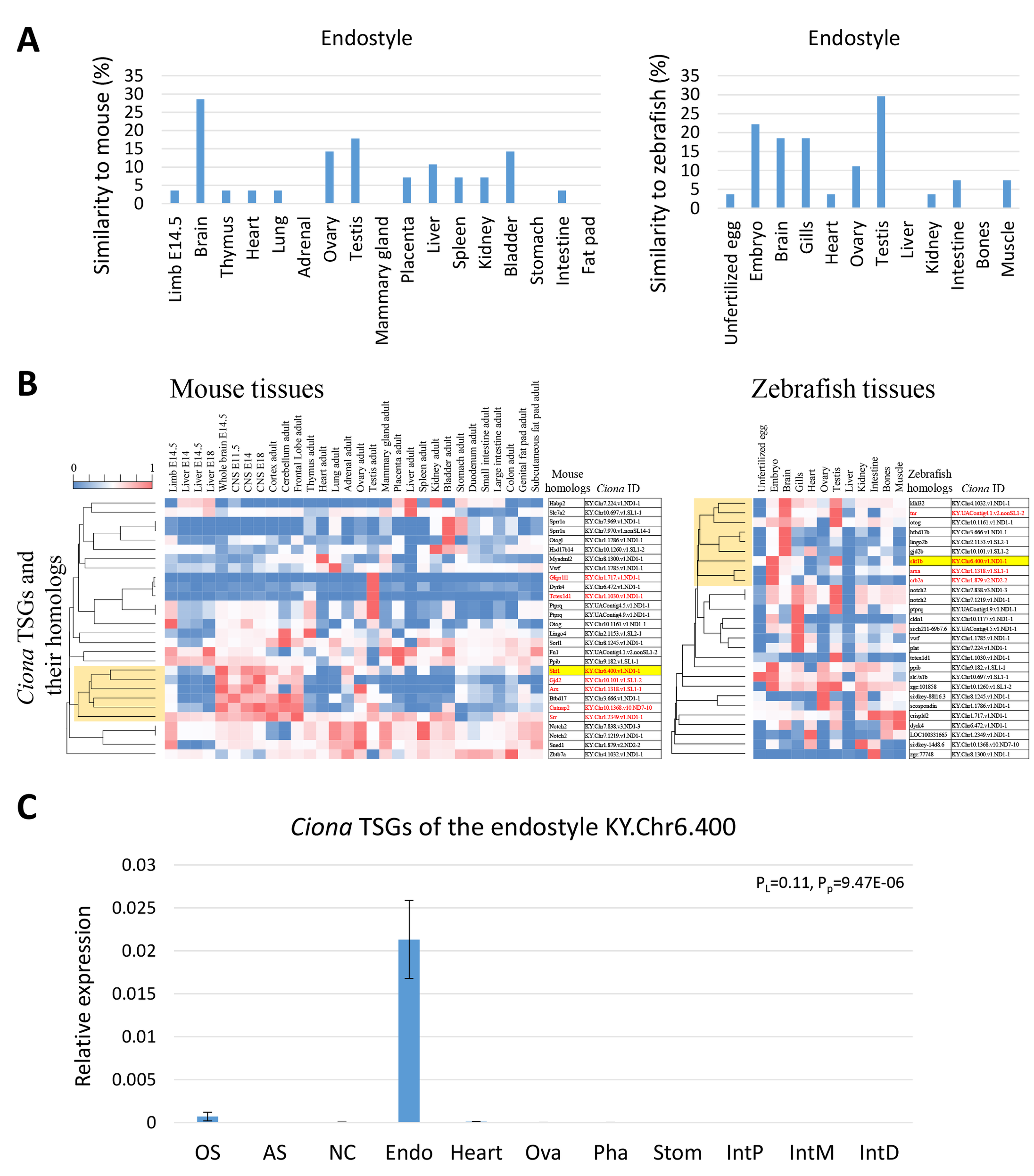

Supplement: S6 Fig — (A) Similarities between Ciona endostyle and mouse (left) or zebrafish (right) tissues were calculated as in Fig 3A. (B) Clustering by tissue distribution of the homologous genes in mice and zebrafish. The heat maps are shown as in Fig 3B. The clusters of highly expressed genes in the vertebrate brain are shown in orange. (C) The endostyle-specific expression of Ciona TSGs in the endostyle was confirmed by qRT-PCR (n = 3–4). Data are presented as in Fig 3C. (TIF) [file pone.0254308.s006.tif]

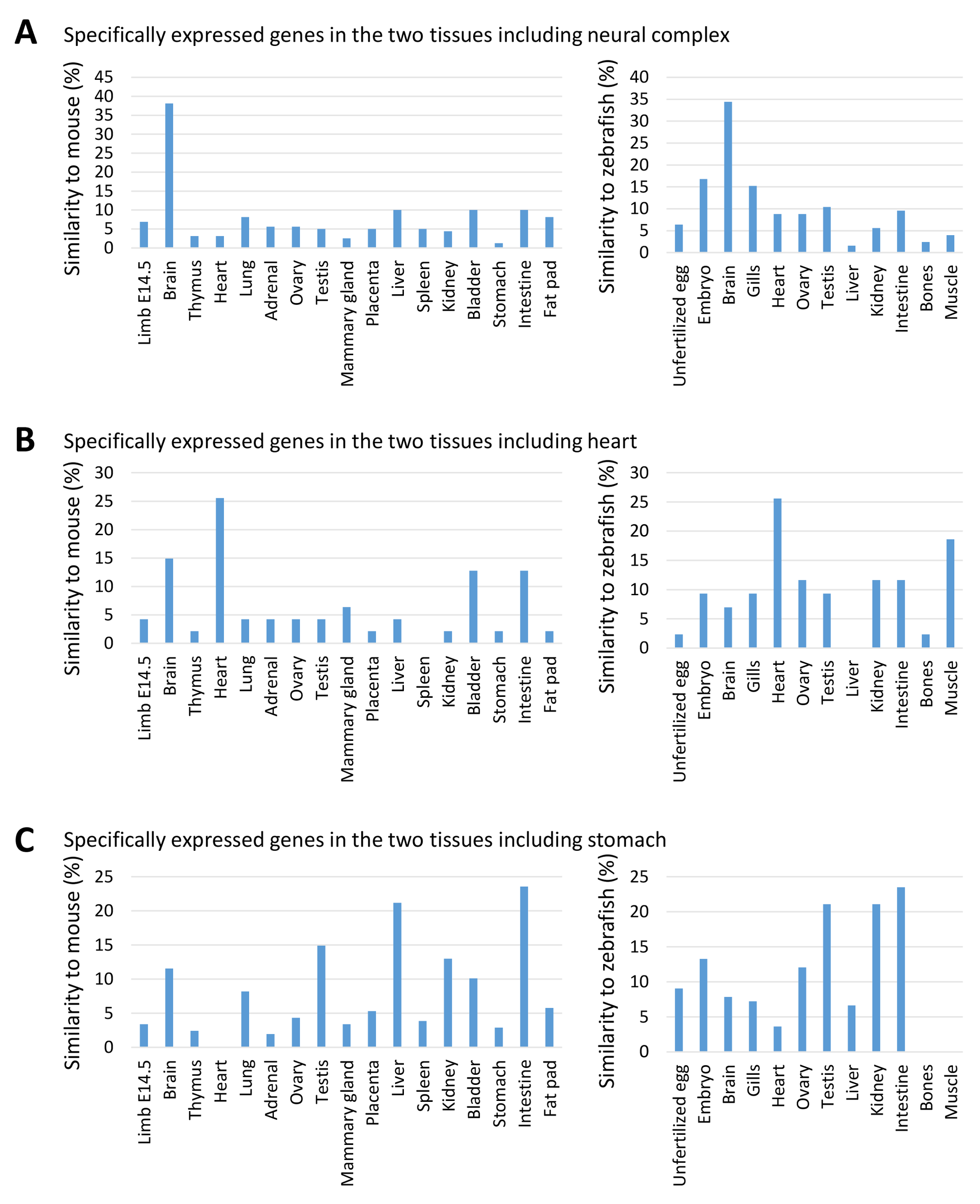

Supplement: S7 Fig — (A) In addition to the 56 Ciona TSGs in the neural complex, specifically expressed genes in the two tissues including neural complex were considered; 234 Ciona genes were screened and the blast hits of 124-vertebrate homologs were analyzed as in Fig 3A. The mouse (38.1%) and zebrafish (34.4%) homologs of Ciona neural complex-specific genes showed high expression in the corresponding mouse and zebrafish brains. Similar analyses were performed on the Ciona heart (B) and stomach (C). (B) In addition to the 31 TSGs in the Ciona heart, 22 vertebrate homologs of the Ciona heart- and the other one tissue-specific genes were analyzed. Mouse (25.5%) and zebrafish (25.6% and 18.6%) homologs were highly expressed in the mouse heart and the zebrafish heart and muscle, respectively. (C) In addition to the 23 TSGs in the Ciona stomach, 200 vertebrate homologs of the Ciona stomach- and the other one tissue-specific genes were analyzed. Mouse homologs were highly expressed in the intestine (23.6%) and liver (21.2%), and zebrafish homologs were expressed in the intestine (23.5%), kidney (21.1%), and testis (21.1%). (TIF) [file pone.0254308.s007.tif]
