# Supplementary material for: Comparative analysis of transcriptomic profiles among ascidians, zebrafish, and mice: Insights from tissue-specific gene expression
Source: PLoS One. 2021 Sep 24;16(9):e0254308. doi: 10.1371/journal.pone.0254308 (PMC8462739; doi:10.1371/journal.pone.0254308)
Supplement: Attachment — Submitted filename: Response to Reviewers.docx [file pone.0254308.s012.docx]

**Point-by-point responses to comments.**

Comments of the academic editor are displayed in italics. Our responses in bold follow each comment.

**Response to the academic editor**

*ACADEMIC EDITOR comments:*

*This is systematic study comparing the gene expression profiles in adult Ciona robusta organs to those in the organs of zebra fish and mice. They authors obtained a new adult Ciona organ RNAseq dataset and used clear and strict criteria to first identify tissue specific genes (TSGs) in Ciona. They then proceed to investigate the expression levels of the orthologs of these TSGs in mice and zebrafish organs based on existing RNAseq datasets. Striking similarities of expression profiles were observed for brain and heart, while similarities for other organs were less apparent or almost non-existing. Based on this the authors concluded that the molecular machineries of some organs are highly conserved (brain and heart), while others are either species specific (e.g. ovary) or spread out over several organs (Ciona stomach shows similarty to mouse/zebra fish liver and other metabolic organs). Overall, this study is a useful resource for scientists with an interest in the evolution of organ systems, in particular at the transition from invertebrates to vertebrates.*

*There are three minor concerns that should be addressed by making respective changes to the manuscript:*

*1.　What is missing in this manuscript is some information whether (and how many) of the orthologs of Ciona TSGs would also fulfil the very same TSG criterion in one or both of the two vertebrates – this could be easily tested and would strengthen the central role of individual genes in these tissues.*

**Thank you for your constructive comment. We have investigated the tissue specificity of the vertebrate homologs of *Ciona* TSGs and found that some vertebrate homologs were also tissue-specific. According to the results, we have revised Fig. 2, S2 Table, and the corresponding parts of the main text (lines 161-171, 233-240, 247-249, 252-254, 281-283, 300-301, and 359-360) and figure legend (lines 154-156) in the revised manuscript.**

*2.　It would be interesting to see whether any of the TSGs is a transcription factor as those would be the drivers of tissue specific gene expression. Thus I recommend to look for this among the TSGs and the expression of the orthologs in mice and zebra fish.*

**We have searched the tissue-specific transcription factors in the *Ciona* tissues and investigated the tissue distribution of their vertebrate homologs. We have found that several vertebrate homologs of the neural complex-specific transcription factors (*Otp*, *arx*, *Pax6*, *pax7a*, *Pou4f3*, *pou4f4*, *Nr2e1*, and *nr2e1*) were also specific or predominant in the vertebrate brain or embryo. On the other hand, in the other tissues, none or quite a small number of tissue-specific transcription factors and their vertebrate homologs exhibited conserved tissue distribution between *Ciona* and vertebrates. These results suggest that some tissue-specific transcription factors act as drivers of tissue-specific gene expression only in the neural complex (brain) but not in the other tissues. We have included these data in the “TFs” tab of the S2 Table and explained them in the revised manuscript (lines 233-240, 254-255, 283-284, and 312-313).**

*3.　The criteria for TSGs are quite stringent (which is good for clarity), but I was wondering what happens when genes that are highly specifically in two (and not just one) Ciona tissue are considered. Do they findings hold true? No extensive test need to be done on this but some discussion about how the similarities between tissue hold up when less stringent criteria are applied would be of interest for the readers.*

**Thank you for your constructive comment. We have investigated *Ciona* TSGs in less stringent criteria considering specific expression in two tissues and obtained similar results, although slight spreading out of the tissue similarities were observed. We have added the data as S7 Fig and described it in the revised manuscript (lines 397-400 and 742-755).**
